# Multi-Classification and Tree-Based Ensemble Network for the Intrusion Detection System in the Internet of Vehicles

**DOI:** 10.3390/s23218788

**Published:** 2023-10-28

**Authors:** Wanting Gou, Haodi Zhang, Ronghui Zhang

**Affiliations:** 1 China Telecom Research Institute, Guangzhou 510630, China; zhanghaodi@chinatelecom.cn; 2 Guangdong Provincial Key Laboratory of Intelligent Transport System, School of Intelligent Systems Engineering, Sun Yat-sen University, Guangzhou 510275, China; zhangrh25@mail.sysu.edu.cn

**Keywords:** intrusion detection system, cybersecurity, machine learning, multiclass classification, data balancing, Internet of Vehicles, Internet of Things

## Abstract

The Internet of Vehicles(IoV) employs vehicle-to-everything (V2X) technology to establish intricate interconnections among the Internet, the IoT network, and the Vehicle Networks (IVNs), forming a complex vehicle communication network. However, the vehicle communication network is very vulnerable to attacks. The implementation of an intrusion detection system (IDS) emerges as an essential requisite to ensure the security of in-vehicle/inter-vehicle communication in IoV. Within this context, the imbalanced nature of network traffic data and the diversity of network attacks stand as pivotal factors in IDS performance. On the one hand, network traffic data often heavily suffer from data imbalance, which impairs the detection performance. To address this issue, this paper employs a hybrid approach combining the Synthetic Minority Over-sampling Technique (SMOTE) and RandomUnderSampler to achieve a balanced class distribution. On the other hand, the diversity of network attacks constitutes another significant factor contributing to poor intrusion detection model performance. Most current machine learning-based IDSs mainly perform binary classification, while poorly dealing with multiclass classification. This paper proposes an adaptive tree-based ensemble network as the intrusion detection engine for the IDS in IoV. This engine employs a deep-layer structure, wherein diverse ML models are stacked as layers and are interconnected in a cascading manner, which enables accurate and efficient multiclass classification, facilitating the precise identification of diverse network attacks. Moreover, a machine learning-based approach is used for feature selection to reduce feature dimensionality, substantially alleviating the computational overhead. Finally, we evaluate the proposed IDS performance on various cyber-attacks from the in-vehicle and external networks in IoV by using the network intrusion detection dataset CICIDS2017 and the vehicle security dataset Car-Hacking. The experimental results demonstrate remarkable performance, with an F1-score of 0.965 on the CICIDS2017 dataset and an F1-score of 0.9999 on the Car-Hacking dataset. These scores demonstrate that our IDS can achieve efficient and precise multiclass classification. This research provides a valuable reference for ensuring the cybersecurity of IoV.

## 1. Introduction

**Figure 1:**
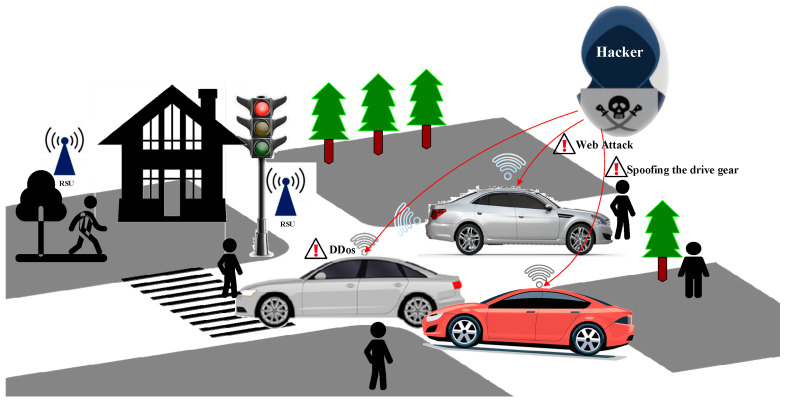
The attack scenario of IoV. There are multiple cyber-attack categories in IoV including denial-of-service (DoS) attacks, web attacks, sniffing, and so on.

Industry 4.0 is undergoing a critical phase of development, with the Internet of Things (IoT) playing an essential foundational role. IoT facilitates intelligent perception, recognition, and management through the interconnection between objects and objects, and between individuals and objects. In recent years, intelligent transportation systems (ITSs) and intelligent vehicles (IVs) have been the research focus of many researchers and technology companies. Through vehicle-to-everything technology (V2X), the internet, inter-vehicle network, and IoT network can be connected to the IVNs of intelligent connected vehicles (ICVs) to form a complex vehicle communication network. However, the increased cyber-attacks against IoV have raised concerns regarding the stability and robustness of IoV. These situations can result in serious consequences such as vehicle unavailability or traffic accidents. The assaults on IoV can be categorized into two primary types, contingent upon the specific target of the attackers: inter-vehicle attacks and intra-vehicle attacks. Figure 1 illustrates the attack scenario of the Internet of Vehicles.

Specifically, IVNs are responsible for the information interaction between the ICVs and the external environment, the vehicle itself, as well as the vehicle and its occupants. The Controller Area Network (CAN) serves as a crucial foundational control network for vehicles in IVNs. It is primarily used to transmit the status information and control information of vehicles, thereby guaranteeing the vehicle’s safe functioning. However, the concision of CAN messages, coupled with the absence of authentication and encryption mechanisms, makes vehicles susceptible to hacking attacks. Numerous researchers conducted experiments to test for vulnerabilities, and proposed corresponding solutions, particularly in the context of the Internet of Vehicles (IoV). For instance, Ref. [1] focused on measuring and exploiting the intervals between periodic in-vehicle messages to fingerprint electronic control units (ECUs) within vehicles. Building on this foundation, they successfully detected abnormal shifts in identification errors. Additionally, another research effort [2] demonstrated the physical feasibility of a long-range wireless attack using a real vehicle and a malicious smartphone application within a connected car environment. As a countermeasure aligned with current Controller Area Network (CAN) specifications, they introduced a security protocol for CAN. Charlie Miller and his team initiated their investigations into the vulnerabilities and attacks within intelligent vehicle systems back in 2013 [3], conducting thorough security risk analyses. Furthermore, with the advent of the IoT, vehicles have become more susceptible to attacks originating from the internet and malicious software such as SolarWinds Orion (version 2019.4-2020.2.1). Notably, another study [4] delved into the analysis of threats posed to IoVs and introduced various methods for targeting intelligent vehicle systems. These methods included interference with braking systems and control over flameout procedures, which, if exploited, could result in remote manipulation and damage to vital vehicle subsystems. Ref. [5] successfully conducted a remote intrusion into the entertainment system of Jeep Cherokee in 2015, which allowed them to manipulate the car and perform dangerous operations like rushing down a roadside slope. Similarly, in September 2016, Keen Lab successfully cracked the in-vehicle central control of Tesla and achieved long-distance remote control up to 12 miles away, leading to sudden braking of a Model S car [6]. Malicious nodes can result in drastic consequences such as loss of life, energy, and money. Additionally, the network is also susceptible to traditional attacks (eavesdropping, sniffing, and others). The mentioned cyber threats may compromise the stability of IVs, resulting in vehicle unavailability or traffic accidents. Therefore, implementing an IDS becomes essential to improve the cybersecurity protection capability of IoV.

IDSs are effective in protecting IoV systems and ICVs against cyber threats. However, the nature of network traffic data is predominantly normal with rare attack instances. It often leads to class imbalance, significantly impacting IDSs’ performance. In addition to class imbalance, the vast quantities of information and the diversity of cyber-attacks continue to render intrusion detection a challenging task. Traditional IDSs mostly rely on established feature libraries and rules to identify known attacks, but this approach is susceptible to evasion. Consequently, IDSs based on machine learning (ML) techniques have gained prominence. ML-based IDSs frame the problem as a classification task, aiming to distinguish between various cyber-attacks and benign network traffic. In this landscape, numerous ML-based techniques have emerged as promising solutions. For instance, random forest (RF) and decision tree (DT) are popular choices for network intrusion detection, as evidenced in studies [7,8,9,10]. Ref. [7] utilized the DT algorithm for detecting both misuse-based and anomaly-based attacks. Meanwhile, in [8], researchers proposed an IDS model that leveraged a combination of ML algorithms within the Spark framework, enhancing intrusion detection capabilities. In another study [9], the Bortua feature selection algorithm was employed to extract vital features from the dataset, resulting in improved classifier performance. Moreover, Ref. [10] introduced an IDS model incorporating principal component analysis (PCA) for dimensionality reduction and a RF classifier for accurate classification. These examples underscore the growing prominence of ML-based IDSs in addressing the intricate challenges of intrusion detection. However, current ML-based IDSs mainly conduct binary classification but perform poorly on multi-classification tasks. And this limitation is accompanied by elevated false positive rates. Given the scale of traffic data in live networks, high false positive rates can result in significant time costs for security analysts. 

In order to conduct more precise multi-classification, enabling accurate and efficient identification of various cyber-attacks in the vehicle communication network, this paper proposes an IDS based on an adaptive tree-based ensemble network for IoV. In our paper, the adaptive tree-based ensemble network (ATBEN) serves as the intrusion detection model. It uses several different ML models as base estimators and stacks them in each layer, which increases the model diversity. Furthermore, the connections between layers are achieved in a cascading manner. We summarize the key contributions of this paper as follows:

1. We investigate the advantages offered by different data balancing approaches in enhancing the IDS performance. Furthermore, we employ a hybrid approach combining the Synthetic Minority Over-sampling Technique (SMOTE) and RandomUnderSampler to achieve a balanced class distribution, effectively addressing class imbalance concerns. We validate the efficacy of this approach by using the CICIDS2017 [11]. Moreover, we use an ML-based approach for feature selection to reduce feature dimensionality, substantially alleviating the computational overhead.
2. We propose an adaptive tree-based ensemble network as the intrusion detection engine of the IDS. It primarily conducts an accurate and efficient multiclass classification of network traffic data originating from the IVNs and external network. This intrusion detection model employs a deep-layer structure, wherein diverse ML models are stacked as layers, and are interconnected in a cascading manner. This model enables the precise and efficient identification of various cyber-attacks, thus safeguarding both IoV systems and ICVs against a variety of cyber threats.
3. We assess the IDS utilizing two datasets: the CICIDS2017, which is widely recognized in network intrusion detection, and the Car-Hacking [12] dataset sourced from the realm of IoV security. The experimental results involve a comprehensive comparison against the prevailing state-of-the-art techniques. The proposed IDS achieved impressive results with an F1-score of 0.965 on the CICIDS2017 dataset, and gave a 0.9999 F1-score on the Car-Hacking dataset. These outcomes illustrate the superiority for multiclass classification on various cyber-attacks both from the external network and the IVNs in IoV.

The rest of the paper is structured as follows. First, Section 2 presents an overview of current research on intrusion detection in IoV. Afterward, Section 3 delves into the methodology employed in this paper. This includes detailing the system design, data preprocessing, and the intrusion detection model of the efficient IDS presented in this paper. In Section 4, we comprehensively examine and analyze the outcomes of the experiments conducted. Finally, a concise summary of the overall conclusions drawn from this research and the plan for future work are provided in Section 5.

## 2. Related Work

The distinctive attributes of IoV, including rapid mobility and foreseeable node movements, pose a security challenge, since its decisions can be a matter of life and death. Consequently, the cybersecurity of the Internet of Vehicles has attracted widespread attention.

With the development of IoV, there has been an increasing number of IVs, which implies a significant rise in communication nodes of IoV. Emerging security threats targeting these communication nodes are highly likely to be exploited, bringing a substantial threat to the cybersecurity of IoV. Efficient and accurate intrusion detection mechanisms must be implemented. ML techniques are extensively employed in the creation of systems for detecting unauthorized infiltrations and have evolved into a mature and effective solution. For instance, Ref. [13] proposed the CSV-ISVM algorithm, which introduced the concept of candidate support vectors (CSVs) to increase the training data size, and also presented a support vector selection approach for the IDS. However, it is essential to note that the proposed half-partition strategy cannot actually be implemented in methods other than binary classification. Ref. [14] combined genetic algorithms with KNN for intrusion detection and performed experiments for multiclass classification tasks. Nevertheless, their experiments encountered challenges related to slow training and high memory requirements when handling large datasets. During their experiments, the dataset was reduced to only thousands of records with several features. Intriguingly, Ref. [15] proposed an interesting aspect, that is, to consider a hybrid approach and employ K-Medoid clustering and SVM feature selection to generate an efficient training set for the Naïve Bayes classifier to accomplish the classification. However, the paper also emphasized the need for more research in determining optimal cluster numbers and initial cluster medoids. Moreover, a series of studies are focusing on the ML-based IDSs that adopt a distributed architectural approach [16,17,18,19,20,21,22,23]. Some of these are studied primarily in fog environments. They generally employ models on fog nodes to improve the intrusion detection’s accuracy. Ref. [16] proposed a DT-based IDS in a fog environment, which can fully detect four kinds of attacks and twenty-two other kinds of attacks. However, this work focused on proving the IDS system’s suitability for big data environments, which may not be directly applicable to resource-constrained IoV contexts. Ref. [17] concentrated on LSTM networks that originate from recurrent neural networks, but the topic of this paper is related to attacks exploiting vulnerabilities of wireless communications, rather than on various cyber security issues. Ref. [18] discussed the increasing risk associated with the growing number of devices connected to IoT. The proposed model in this study, however, required substantial datasets and extended training periods, making it less suitable for real-time intrusion detection within the IoV context. Ref. [19] implemented network monitoring of IoT devices through network packet analysis. However, this solution necessitated a central location to provide the IDS with a comprehensive view of the entire IoT network. This approach may not be directly transferable to the IoV, where nodes are highly mobile and dispersed. Ref. [20] presented a cooperative adaptive network IDS framework, focusing on cooperation-based detection architecture using online machine learning algorithms. However, the applicability of this model to the IoV context remains uncertain due to its distinct characteristics. On the other hand, there are also distributed IDSs based on federated learning [22,23], a technique that aligns with distributed architectures and emphasizes user privacy. Ref. [22], for instance, introduced a federated machine-learning-based IDS model that trains IoT device models using local data while preserving sensitive IoT information security. Furthermore, federated learning-based server aggregation with local models maximizes client detection efficiency. Another approach, presented in [23], employed federated learning in conjunction with a long short-term memory (LSTM) framework to develop an intrusion detection method (FL-LSTM). This method addressed privacy concerns and underwent testing against a modified dataset of system calls from AT&T, although the dataset’s age may limit its representativeness in contemporary devices.

Deep learning (DL), a subset of machine learning, has outstanding performance in NLP and CV in decades. Several commonly employed DL algorithms for IDS include CNN, RNN, and gated recurrent unit (GRU). These DL algorithms are generally recognized for their superior efficiency when compared to traditional machine learning techniques. Ref. [24] presents a comprehensive overview of DL approaches for cyber security intrusion detection, encompassing the datasets used and a comparative analysis. Ref. [25] proposed a lightweight dense random neural network (DnRaNN) tailored for intrusion detection within the IoT. Additionally, it proposes integrating a field-programmable gate array (FPGA)-based accelerator with DnRaNN to optimize detection algorithm performance. The utilization of CNN is somewhat more intricate compared to other DL algorithms, as it necessitates data to be structured as image-like matrices. This requirement entails data normalization and transformation into a matrix form [26]. Ref. [27] presented a stacked DL approach for detecting malicious attacks in SCADA systems. It compared multiple ML models and found that XGBoost outperformed others due to its robustness and feature invariance. Surprisingly, DL models did not perform as well in this context. Ref. [28] introduced CPS-GUARD, an intrusion detection approach focused on cyber-physical systems (CPSs). It utilized a single semi-supervised auto encoder with an outlier-aware threshold-setting technique, which yielded high recall, precision, and low false positive rates in detecting intrusions. While CPS-GUARD’s performance in the context of CPSs was commendable, its direct comparison with IoV may have been limited due to the differing nature and requirements of these two domains. In [29], LSTM was employed as a sub-module to enhance time series classification for fully convolutional networks (FCNs). Additionally, an attention mechanism was introduced, resulting in the improved performance of the model in intrusion detection. There remains room for further exploration regarding why the attention LSTM cell sometimes underperforms the general LSTM cell on specific datasets. Moreover, expanding the proposed models to accommodate multivariate time series has been largely unexplored in this context. Additionally, some researchers have integrated deep learning techniques with SVM. They combined the one-dimensional convolutional auto encoder (1D CAE) and the one-class support vector machine (OCSVM) into a one-stage model [30]. Experimental results demonstrated the model’s excellent detection performance and generalization performance. Another approach, described in [31], integrated k-means, DNN, and SVM to create a two-stage model. Initially, k-means clustering was employed to identify anomalies, and subsequently, intrusion detection was performed on the clustered data using a combination of DNN and SVM. The experimental results demonstrated the model’s robust ability to accurately detect targets. However, it did exhibit certain limitations when it came to generalization performance. 

While the aforementioned IDS solutions are designed for general networks, there has been a noticeable increase in attention towards the development and research of IDS specific to the IoV domain in the past few years. For intra-vehicle attack detection, Ref. [12] proposes an IDS based on a deep CNN to optimize the flow data of the CAN bus. They use datasets constructed from real vehicles to evaluate the model, giving an excellent result. Nevertheless, the average in-vehicle network infrastructure generally lacks sophisticated CPUs, let alone GPUs, which implies that implementing the proposed IDS within vehicles would necessitate additional hardware. Ref. [32] introduces a novel framework, named DPFL-F2IDS scheme, for an edge inter-vehicle network that transmits basic safety messages, consisting of differentially private federated learning (DPFL) and F2IDS (framework for IDS). DPFL-F2IDS aims to prevent member inference attacks common in standard federated learning, but it grapples with the challenge of striking a balance between utility and privacy metrics. Ref. [33] simulates vehicular ad hoc network (VANET) attack scenarios, collects network traffic data, and performs traffic analysis based on statistical methods. The proposed IDS determines whether to accept or reject upcoming data based on flow analysis, but the approach is less accurate when multiple malicious events occur. Ref. [34] focuses on possible attacks on autonomous vehicles communicating with the outside world via VANETs, utilizing neural networks to distinguish denial-of-service attacks. In addition, some researchers consider that the multi-dimensionality of network traffic data features may lead to the high complexity of the detection model. Ref. [35] uses PCA technology for feature dimensionality reduction, and then constructs a cyber-attack classifier by using a low-parameter deep neural network to detect anomaly. However, PCA is a linear static model and may not effectively capture the nonlinear and dynamic aspects of the data. Using PCA in this context could potentially result in information loss. Finally, it is noted that ensemble approaches are effective solutions for the classification task of network traffic data, which has a class imbalance problem, and many studies have used ensemble technology to design IDS and achieve outstanding detection performance [36,37]. Ref. [36] introduces an intelligent IDS founded on tree-structured machine learning models. The results gleaned from implementing this IDS on standard datasets underscore its ability to identify various cyber-attacks within AV networks. The research presented in [37] illustrates ensemble learning-based network intrusion detection systems (NIDS) that adeptly utilize various individual ML models to make informed estimations. These two studies lay the groundwork for our proposal. However, we have undertaken further efforts to enhance the performance of our IDS.

It can be observed that recent research on IDS has varied focuses. For example, Refs. [7,8,9,10,13,14,15,16,24,25,26,27,28,29,30,31] design IDSs for general networks, Refs. [17,18,19,20,21,22,23] concentrate on the distributed deployment of IDSs in IoT and IoV, and Refs. [32,33,34,35,36,37] are predominantly concerned with cybersecurity in IVNs. We give a comparison of the literature included in this section in Table 1. However, most current IDSs tend to perform binary classification and struggle with multiclass classification tasks. Additionally, there exists serious imbalance in network traffic data, presenting a challenge for multiclass classification. Therefore, we seek to develop an efficient and robust IDS that can mitigate the impact of class imbalance and further enable accurate, efficient, and fine-grained identification of various cyber-attacks in IoV.

## 3. Methodology

### 3.1. System Design

**Figure 2:**
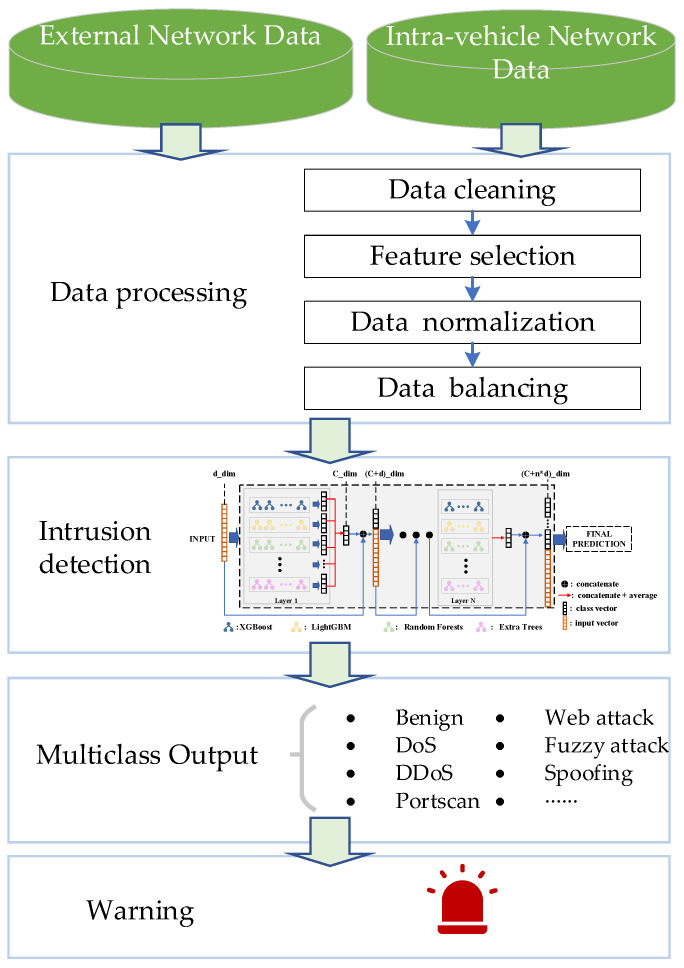
The workflow of intrusion detection.

In order to achieve an accurate, efficient, and fine-grained classification of network traffic in the vehicle communication network, protecting ICVs in IoV against cyber-attacks from external networks and IVNs, we propose an efficient IDS based on an adaptive tree-based ensemble network (ATBEN). Figure 2 illustrates the comprehensive workflow of this IDS. Figure 2 depicts the four components of the network IDS: (1) data collection, (2) data processing, (3) intrusion detection engine, and (4) detection result output. We aim to enhance the security of ICVs by implementing a robust IDS that effectively identifies and mitigates potential threats.

### 3.2. Data Processing

#### 3.2.1. Data Collection

The foundation of any IDS lies in the collection of comprehensive and ample data samples, pivotal for training and testing intrusion detection models. Training intrusion models requires sufficient network traffic data captured under both benign and malicious conditions. The collected data are segregated into two distinct sets for the proposed IDS: (1) a training set utilized to model training, and (2) a testing set for evaluating model performance.

#### 3.2.2. Data Cleaning

To facilitate a robust evaluation of the IDS performance, a number of network intrusion detection datasets have been available for related research. Among them, there are several well-known datasets such as KDD 99, NSL-KDD [38], UNSWNB15 [39], and CICIDS2017 [11]. Concurrently, collaborative efforts by researchers have led to the provisioning of dedicated car-hacking datasets to promote automotive safety research in IoV [12]. However, in order to utilize these data more efficiently for model training and testing, a series of data cleaning steps must be carried out. For example, we need to check the dataset for missing values, constant values, and other outliers that cannot be used for model learning. These anomalies necessitate either cleaning or transformation into valuable data.

#### 3.2.3. Feature Selection (FS)

Intrusion detection datasets typically contain a range of the general network attributes. For example, the CICIDS2017 dataset covers 78 network attribute characteristics. In general, datasets with multiple characteristics fall into the realm of high-dimensional data. We usually use some feature selection techniques to preserve the critical attributes of data while filtering out noise and insignificant features. This process reduces data redundancy and computing overhead. First, we calculated the correlation among the 78 features in the CICIDS2017 dataset using the equations below. According to the calculated correlation coefficient matrix, 27 highly correlated features were removed from the dataset.

(1) $$\rho_{X,Y}=\frac{cov\left(X,Y\right)}{\sigma_{X}\sigma_{Y}}$$

(2) $$cov\left(X,Y\right)=\frac{1}{N-1}\sum_{i=1}^{N}\left(X_{i}-\bar{X}\right)\left(Y_{i}-\bar{Y}\right)$$

(3) $$\sigma_{X}=\sqrt{n\sum X_{i}^{2}-{\left(\sum X_{i}\right)}^{2}}$$

(4) $$\sigma_{Y}=\sqrt{n\sum Y_{i}^{2}-{\left(\sum Y_{i}\right)}^{2}}$$

(5) $$\bar{X}=\frac{1}{N}\sum_{i=1}^{N}X_{i}$$

(6) $$\bar{Y}=\frac{1}{N}\sum_{i=1}^{N}Y_{i}$$

(7) $$X=\left\{X_{i}|i=1,2,\cdots \right\},\;Y=\{Y_{i}|i=1,2,\cdots \}$$

where X and Y are the sets of samples, $\bar{X}$ is the mean of X, $\bar{Y}$ is the mean of Y, σ denotes variance, and $cov\left(X,Y\right)$ is the covariance of X and Y.

At the same time, we further explored different approaches for data dimensionality reduction, reducing the dimensional feature space. PCA was one of our choices, which has been extensively employed. Besides employing PCA, we also utilized ML-based approaches for feature selection to reduce feature dimensionality. We used two different ML models (random forest and XGBoost) to calculate the importance of each feature. The output importance scores of these models were averaged and sorted in descending order. We then selected features in the order of their importance until the cumulative importance reached a threshold of 95%. Finally, any remaining features were discarded. 

**Figure 3:**
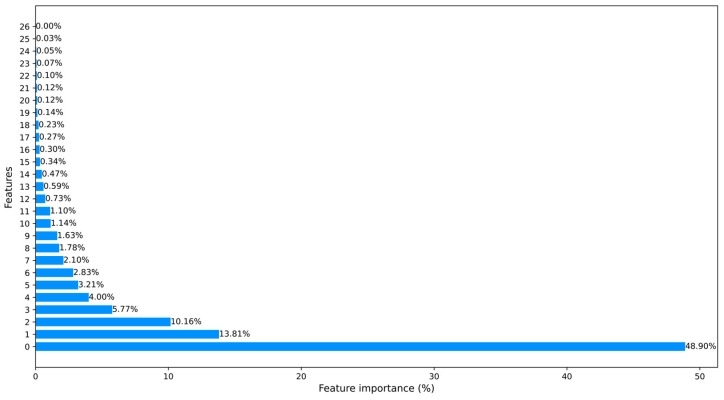
The processed CICIDS2017 dataset ranked features using the ML-based FS method.

**Figure 4:**
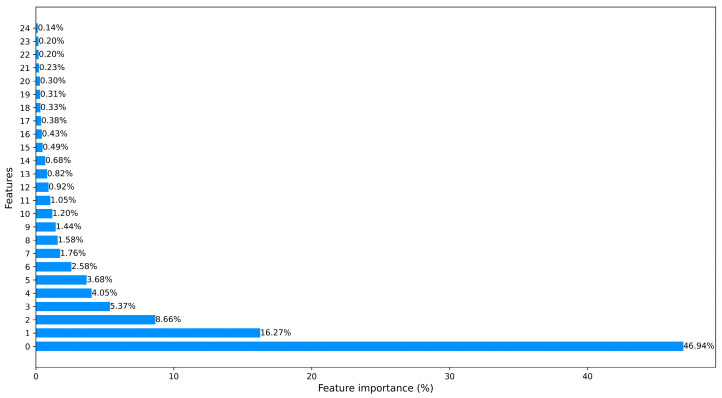
The processed CICIDS2017 dataset ranked features using PCA.

**Figure 5:**
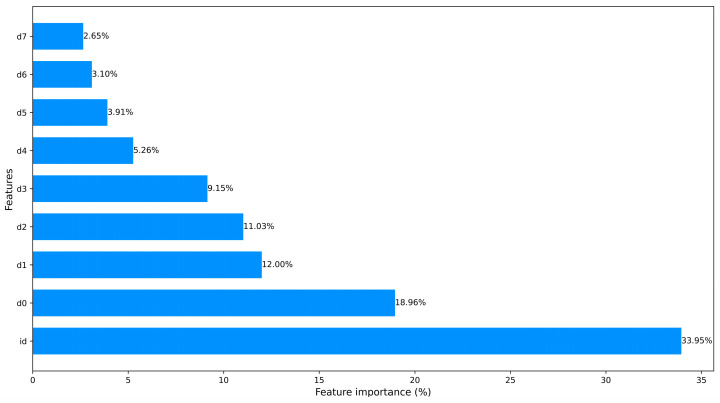
The Car-Hacking dataset ranked features.

These approaches significantly help to save training time and computational resources. In Figure 3 and Figure 4, we counted the feature importance percentages after using the PCA and the ML-based FS method. Since Car-Hacking data only have 8 features, it obviates the need to carry out dimensionality reduction. The importance percentages of data features are shown in Figure 5.

#### 3.2.4. Data Normalization

Normalizing high-dimensional data is crucial for data preprocessing. Unnormalized high-dimensional data can lead to an increased computational burden on ML models, slowing down their learning and effectiveness. This paper employs the quantile transformation for data normalization. The quantile transformation is a technique that utilizes non-linear transformations to normalize data, and it demonstrates robustness against outliers. Specifically, it involves estimating a function that fits input variables as much as possible, and transforming the values into a consistent distribution ranging from 0 to 1. Subsequently, the acquired values undergo a conversion using appropriate quantile functions to achieve the desired distribution.

(8) $$y=G^{-1}\left(F\left(x\right)\right)=G^{-1}\left(\int_{\infty }^{x}f_{x}\left(t\right)dt\right)$$

where x denotes the features, $F\left(x\right)$ denotes cumulative distribution function of features, and $G^{-1}$ represents the quantile function of the distribution for expected output values.

#### 3.2.5. Data Balancing 

Based on current open-source IDS datasets, we can observe that these datasets tend to exhibit pronounced data imbalances. For example, both NSL-KDD and CICIDS2017 manifest a substantial majority of benign samples, accounting for 95% and 90%, respectively, with the remaining samples encompassing various types of attacks. This is mainly due to the fact that instances of attack states in real-life scenarios are relatively rare, and most of the time the network remains in its regular state. The high imbalance of the dataset causes classifier performance to tip in favor of the majority class (benign), making classifying minority classes very challenging. Multiple data balancing techniques have been proposed to overcome it. Common methods used to address class imbalance include under-sampling, over-sampling, class weight strategy, and sample weight strategy. Random oversampling balances the data by randomly copying samples of the minority classes but is prone to overfitting. The SMOTE used in this paper is an oversampling technique to enrich the data by synthesizing minority samples. It utilizes the concept of K-nearest neighbors to analyze the minority class and generate high-quality new samples to expand minority classes in the dataset. In addition, we used under-sampling to decrease the count of instances in the majority class. The principle behind under-sampling is to create a more balanced distribution between the less represented and more prevalent classes. This is accomplished based on the method of randomly choosing a smaller part of instances from the majority class.

At the same time, this paper also considers class weight strategy and sample weight strategy as two methods for adjusting sample weights. Class weights are special cases of sample weights, which give the same sample weights to the same class of samples. For example, most classifiers in the sklearn library provide parameters for setting sample weights. Therefore, we explored the gains brought by several data balancing techniques to intrusion detection models as an important work in this paper.

### 3.3. The Proposed Intrusion Detection Model

#### 3.3.1. The Inspiration

Ensemble learning techniques [40] have a natural advantage when it comes to dealing with highly imbalanced classification problems. This can be observed from the winning algorithms in competitions such as KDDCup, Netflix competition, Kaggle, and others over the past few decades, where ensemble techniques have been widely utilized. Therefore, ensemble learning techniques serve as the fundamental approach in constructing our intrusion detection model. 

The impressive performance of deep learning in recent years has provided us with valuable insights. The success of deep learning is due to the construction of its deep structure. After the original data undergoes multi-layer representation learning, it gradually extracts features. Typical algorithms include MLP, CNN, DNN, etc. However, compared to neural network models, tree-based models often exhibit better performance on tabular data. 

#### 3.3.2. The Model Structure

**Figure 6:**
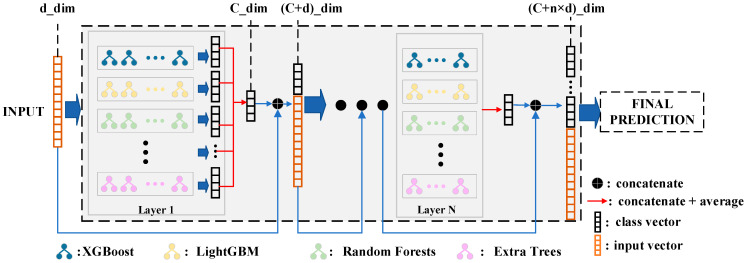
The architecture of the adaptive tree-based ensemble network (ATBEN).

We propose a tree-based network model with adaptive depth. Considering that the strong effect of ensemble learning heavily relies on the diversity of its components, we use various machine learning models (XGBoost [41], LightGBM [42], RF [43], Extra Trees (ET) [44], and so on) as the base estimator, stacking them together as the layers of the network. Then, the connections between the layers are achieved in a cascading manner. However, it is interesting to note that the estimator for each layer can be an ensemble model such as XGBoost, RF, etc. Therefore, our overall model can be considered an ensemble of ensembles (Figure 6).

Similar to deep learning, diversity can help models expand the depth of their network, avoid overfitting, and improve model performance. In machine learning, diversity is also a crucial aspect that supports model learning. In addition to deploying the different estimators in each layer, we also introduce original inputs to enhance data diversity during the training process. It progressively supplements class distribution probability vectors from previous layers, providing prior knowledge for subsequent training. Experimental results have demonstrated that such processing gains in model accuracy.

In machine learning, k-fold cross-validation is commonly utilized for model tuning to find the optimal hyperparameter values that maximize the model’s generalization performance. As mentioned earlier, the collected data related to network traffic is segregated into training purposes and another set reserved for testing. To prevent overfitting during the training process, which may result in poor performance on the testing set, we employ k-fold cross-validation with k set to 5. Specifically, in the training of each layer, the validation samples are drawn from the input data in a percentage of 20%, which are employed to assess the current model performance (we choose recall as the validation evaluation index, and its calculation formula is shown in the next section). If the evaluation metric of the current layer decreases beyond the threshold, the model training is terminated, and the next forest layer is not expanded. Therefore, the depth of the proposed model is adaptive, which means that there is no artificial setting.

#### 3.3.3. ML Models

ML models used in the proposed intrusion detection model involve RF, ET, XGBoost, etc. Most of these models are built upon various rule-based ensemble decision trees. The decision tree (DT) [45] consists of decision nodes and leaf nodes. Decision nodes denote decision directions, while leaf nodes represent the final prediction outputs. Random forest constructs a multitude of decision trees using the bagging technique. The selection of optimal feature split points in random forest is guided by the principle of minimizing the Gini index. The formula for calculating the Gini is shown in Equation (9). Extra trees (ET) is highly similar to random forest, as it is a model that also combines numerous decision trees. What sets ET apart is that it employs all samples and selects features randomly for branching.

(9) $$Gini\left(D\right)=1-\overset{K}{\underset{i=1}{\sum }}{\left(\frac{\left|C_{i}\right|}{\left|D\right|}\right)}^{2}$$

where D is the total number of samples, $C_{i}$ is the number of the i-th class sample, and K is the class number.

XGBoost is a gradient boosting-based algorithm that integrates a multitude of decision trees. It is characterized by its efficiency, flexibility, and lightweight nature. In each iteration, XGBoost only optimizes the sub-model for the current step. For example, in the i-th step, it just takes the $f_{m}\left(x_{i}\right)$ into consideration.

(10) $$F_{m}\left(x_{i}\right)=F_{m-1}\left(x_{i}\right)+f_{m}\left(x_{i}\right)$$

In this equation, $f_{m}\left(x_{i}\right)$ is the current model, and $F_{m-1}\left(x_{i}\right)$ is the model already determined in the previous step.

The objective function of XGBoost is composed of the loss function and the regularization term that constrains the model complexity, as shown in Equation (11). And the regularization term can be obtained according to Equation (12). After optimizing the loss function component and the regularization component, the final objective function is obtained as shown in Equation (13).

(11) $$Obj=\overset{n}{\underset{i=1}{\sum }}L\left(y_{i},\hat{y_{i}}\right)+\overset{t}{\underset{i=1}{\sum }}\Omega \left(f_{i}\right)$$

(12) $$\Omega \left(f_{t}\right)=\gamma T+\frac{1}{2}\lambda \overset{T}{\underset{j=1}{\sum }}\omega_{j}^{2}$$

(13) $$Obj=-\frac{1}{2}\overset{T}{\underset{j=1}{\sum }}\frac{G_{j}^{2}}{H_{j}+\lambda }+\gamma T$$

where T denotes the count of leaf nodes, and $\omega_{j}^{2}$ is the L2-norm of the leaf scores; λ and γ stand for the penalty coefficients, while G and H denote the cumulative first- and second-order gradient statistics of the loss function.

We utilize XGBoost for multiclass classification tasks, where the loss function is softmax as defined in Equation (14).

(14) $$softmax\left(z_{i}\right)=\frac{\exp\left(z_{i}\right)}{\sum_{j}\exp\left(z_{j}\right)}$$

where $z_{i}$ is the i-th sample in data z.

## 4. Experiments

All experiments in this paper were executed based on the Ubuntu 22.04.2 LTS operating system, using PyTorch as the development framework and Python as the development language. The CPU used in the experiment was Intel(R) Xeon(R) Gold 6348 CPU @ 2.60 GHz, and the GPU was NVIDIA A10.

### 4.1. Dataset Description

An IDS for connected car environments should be able to detect various attacks both originating from IVNs and external networks. From that point on, we utilized the CICIDS2017 and Car-Hacking datasets to evaluate the development of the IDS in this paper.

#### 4.1.1. CICIDS2017 Dataset 

**Figure 7:**
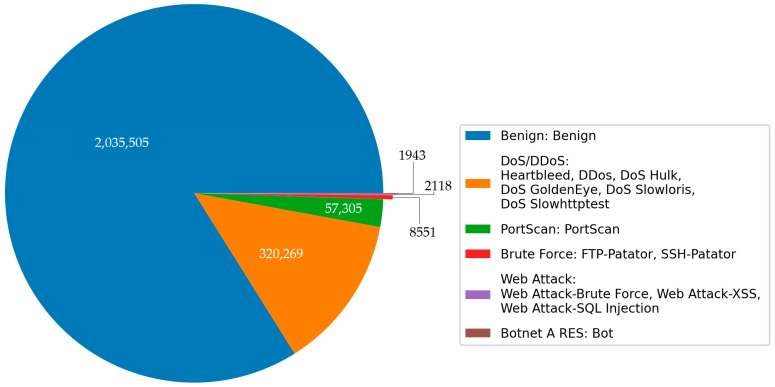
Class distribution of the original CICIDS2017.

Intrusion detection requires a representative network dataset, and the benchmark dataset is the basis for evaluating different IDSs. This dataset is a contemporary flow-based network intrusion dataset. Before the availability of CICIDS2017, popular intrusion detection datasets included KDD99 and NSL-KDD. A list with 11 criteria was published in 2016, outlining the essential requirements for a reliable intrusion detection dataset. CICIDS2017 fulfills all of these criteria, making it a new and comprehensive dataset. The benign data in the CICIDS2017 dataset account for more than 80%, and the attacks implemented included various attack types. This dataset contains thirteen subcategories, which we organized into six main categories when preprocessing data. The detailed information is illustrated in Figure 7. Furthermore, we divided the CICIDS2017 dataset into two parts: one for training and the other for testing. This allocation maintains a proportion of 60% for training the model and 40% for evaluating its performance. The detailed description of attacks mentioned in Figure 7 are provided in Appendix A.

#### 4.1.2. Car-Hacking Dataset

**Figure 8:**
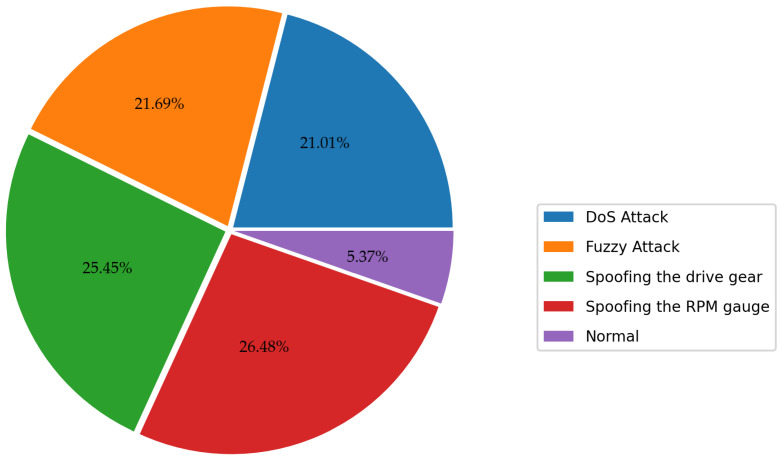
Class distribution of the Car-Hacking dataset.

The Car-Hacking dataset is compiled by recording CAN traffic through the OBD-II port during the occurrence of a CAN attack [12]. The dataset covers 10 features such as timestamp, CAN ID, DLC data bytes, CAN packet, and label (R/T), of which DLC and label provide very limited valid information, so we removed them in the data cleaning stage. Of course, it is also necessary to clear all data anomalies (NAN value, constant value, etc.), as described in Section 3. Since we cleared the label column originally given to the dataset, we had to make a qualified label. This dataset covers benign traffic and four attack categories, such as DDoS, Fuzzy, and Spoofing, so we labeled each data sample with its attack category name. On the other hand, the dataset’s distribution is illustrated in Figure 8. It is clear that the attack samples in this dataset are rich enough, accounting for 95% of the total dataset, so we did not perform data balancing processing on it.

In addition to cleaning the data, we also performed a data shuffling operation to divide the dataset into training and testing sets for intrusion detection model training and testing.

### 4.2. Evaluation Metrics

Network intrusion detection performs the classification, distinguishing between benign traffic and various types of attack behavior. In machine learning, the indicators mentioned below can provide information about the classifier’s performance in various aspects, and the relevant definitions are shown below.

(15) $$Accuracy=\frac{TP+TN}{TP+TN+FP+FN}$$

(16) $$Recall=\frac{TP}{TP+FN}$$

(17) $$Precision=\frac{TP}{TP+FP}$$

(18) $$F1-measure=2\times \frac{precision\times recall}{precision+recall}$$

In particular, a binary classifier can be viewed as classifying instances into positive or negative. An example classified as itself is counted as a true positive (TP) or a true negative (TN). A false positive (FP) or false negative (FN) is recorded if the example is not determined as itself, such as an example which was predicted positive but was negative, or was predicted negative but was positive.

According to the definition in Equation (15), accuracy is used to measure the proportion of data correctly classified. It is important to note that intrusion datasets frequently suffer from class imbalance. This imbalance often leads to commendable accuracy levels in benign traffic classification, but a reduced ability to effectively detect malicious traffic. Thus, we pay more attention to precision, recall, and the F1-measure. A qualified IDS needs to take into account both precision and recall. The F1-measure emerges as a well-rounded evaluation metric by capturing both precision and recall through the calculation of their harmonic average. A higher value of the F1-score signifies superior algorithmic classification prowess. In addition to the values above, another aspect to assess is the execution time. This parameter reveals the time required to process one sample, providing valuable insights into the system’s efficiency. 

### 4.3. Results and Discussion

We aimed to develop an accurate, efficient, and refined IDS to protect intelligent and connected vehicles (ICVs) of IoV from various cyber-attacks. In this section, we utilize the datasets described in Section 4.1 to carry out experimental verification. We evaluate model performance using the previously mentioned metrics and discuss the results from various perspectives.

During the data preprocessing stage, we investigated the advantages offered by different data balancing approaches in enhancing the IDS’s performance by using the CICIDS2017 dataset. We balanced the class distribution of the training set using various methods, without altering the testing set. Table 2 displays the class information of the preprocessed training set.

We evaluated several data balancing approaches based on RF, which can be seen as a preliminary experiment. The balanced training set was used for model training, while the original testing set was employed to assess the model’s performance. The experimental results are displayed in the figure below. 

**Figure 9:**
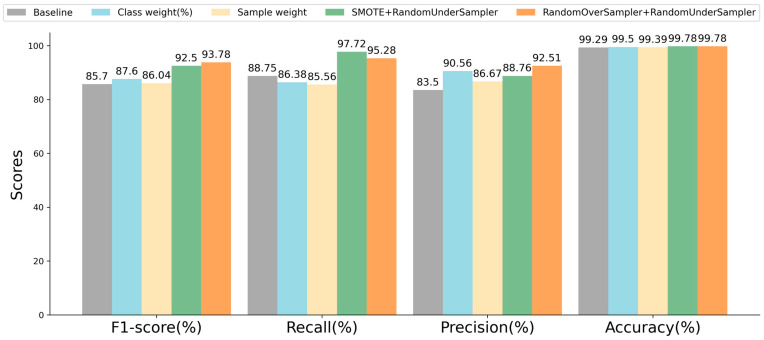
Detection performance of RF using different data balancing techniques.

In Figure 9, the baseline represents the experimental results obtained without employing any data balancing techniques, giving an 85.7% F1-score, 88.75% recall, 83.5% precision, and 99.29% accuracy. There are four data balancing approaches used in the preliminary experiment: (1) class weight strategy, (2) sample weight strategy, (3) a combination of SMOTE and RandomUnderSampler, (4) and a combination of RandomOverSampler and RandomUnderSampler. Figure 9 shows that the employment of these four balancing approaches can significantly enhance the model detection performance. Compared with the baseline, the F1-score improved by 0.34~8.08%, and the accuracy saw an improvement of 0.1~0.49%. Specifically, the two approaches combining oversampling and undersampling yielded the most favorable results. Finally, we decided to employ the approach that combines SMOTE and RandomUnderSampler to balance the original training set.

Table 3 illustrates a comparison of the model detection performance using different approaches to reduce feature dimensionality. Besides employing PCA, we also explored ML-based approaches for feature selection to reduce feature dimensionality. It should be noted that the baseline represents the model detection performance without any feature dimensionality reduction. Our findings revealed that employing feature dimensionality reduction minimally affected the model detection performance while greatly reducing the computational cost. More specifically, when employing PCA, there was a 0.89% decrease in the F1-score, a 1.13% decrease in recall, and a 0.73% decrease in precision. However, it resulted in a substantial reduction in data feature dimension from 49 to 25. Moreover, when using the ML-based method, F1 decreased by 0.89%, recall decreased by 1.13%, precision decreased by 0.73%, and the data feature dimension was reduced from 49 to 27.

To select the base estimator for building the ultimate intrusion detection model, we evaluated various ML models (RF, ET, XGBoost, and LightGBM) using the processed CICIDS2017 dataset. The first four rows of Table 4 display the experimental results, revealing that bagging-based ensemble models like RF and ET outperform boosting-based machine learning models (XGBoost and LightGBM). This superiority is evident in both accuracy and speed. Therefore, we used RF and ET as the base estimators to build the final intrusion detection model. Taking the model diversity into consideration, we used XGBoost as another base estimator. When stacking the different base estimators to build model without extending layers, it gave a 94.77% F1-score, 92.3% recall, 98.66% precision, and 99.88% accuracy.

The last row of Table 4 presents the experimental results of the ATBEN. Compared with the single-base ML model, the ATBEN shows an improvement of 3.09~7.6% in the F1-score, 0.26~13.42% in recall, 0.82~5.76% in precision, and 0.13~0.35% in accuracy. Further comparisons revealed that the expansion of model depth further enhances the effectiveness of the model in terms of detection performance. In contrast to the stacking model, the ATBEN exhibited a 1.69% improvement in the F1-score, a 6.07% increase in recall, and a 0.02% enhancement in accuracy. This compellingly demonstrates that information undergoing layer-by-layer learning contributes to enhancing the model performance. In Table 4, we emphasize the optimal performance data in bold formatting.

**Figure 10:**
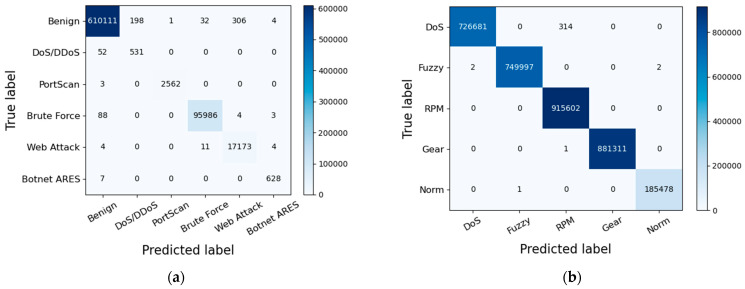
(**a**) The confusion matrix on the testing set of CICIDS2017; (**b**) the confusion matrix on the testing set of the Car-Hacking dataset.

We created confusion matrixes for the proposed model both on the testing set of the CICIDS2017 dataset and the Car-Hacking dataset in Figure 10. The count values assigned to each category offer insights into both accurate and incorrectly classified classifications. It is evident that the proposed model can effectively distinguish between various network traffic samples, whether benign or malicious.

In addition, Table 5 illustrates the different evaluating metrics achieved on different categories in the CICIDS2017 testing set. It can be seen that ATBEN gains high scores (precision, recall, and F1-score) for all categories, almost approaching 100%. This indicates a high success rate in detecting the majority of attacks. The “Botnet” classes, however, have a high recall and a low precision. Network intrusion detection is hoped to detect all malicious activities as much as possible, which means that high recall is important. However, a few false alarms being triggered (low precision) is of relatively lesser concern.

The Car-Hacking dataset was also used to test the proposed IDS. Table 6 displays the experimental results. As evidenced by Table 6, our intrusion detection model provides nearly perfect detection performance across all categories, almost approaching a 100% F1-score. This highlights the exceptional ability of the proposed IDS to identify malicious behaviors within IVNs.

Moreover, we summarize the detection performance of different methods on the two datasets, respectively, in Table 7 and Table 8. And we emphasize the optimal performance data in bold formatting. Most studies in Table 7, such as MLP, 1D-CNN, and LSTM in [46], conduct binary classification rather than multiclass classification. Among them, the 1D-CNN model proved to be the most successful with the highest F1-score of 0.939. Our goal is to ensure that an IDS not only has the ability to differentiate between benign and intrusive behavior, but also to accurately classify data across multiple categories. We compared our method against others shown in Table 7. It is noticeable that our method achieves higher scores in terms of precision, recall, and F1-score, which has reached 0.965. Additionally, we are 2.5% more accurate than DBN [47] in the F1-score, which also performs multiclass classification. It should be mentioned that DeepDFL [48] conducts multiclass classification of 12 classes, while it produces a high precision of 0.948 and a low recall of 0.448. The reason for its inferior performance compared to ours could be attributed to the complexity of the classification task or the model’s design.

Table 8 showcases how our model outperforms other approaches when testing on the Car-Hacking dataset. Our model obtains a remarkable score of 99.99% in the F1-score, recall, precision, and accuracy. The first four rows of Table 8 give the experimental results using different base ML models. Evidently, it can be observed that the F1-score achieved by these ML models is lower than ours. Specifically, our model shows a 0.03~27.53% improvement in the F1-score and a 0.02% to 24.46% improvement in accuracy. Once again, our model structure has been proven to be highly effective. 

Furthermore, our intrusion detection model outperforms the state-of-the-art methods [12,32,49,50] with a significantly higher F1-score ranging from 0.02% to 0.99%. It is also generally higher than other methods in indicators such as recall, precision, and accuracy, showing excellent detection capabilities.

Finally, we conducted a comparison of the existing studies with ours in the table below (Table 9).

## 5. Conclusions

The objective of this paper is to develop an accurate, efficient, and refined IDS to protect intelligent and connected vehicles (ICVs) of IoV from various cyber-attacks. In this paper, we investigate the advantages offered by different data balancing approaches in enhancing IDS performance. Specifically, we employ a hybrid approach combining SMOTE and RandomUnderSampler to achieve a balanced class distribution and demonstrate that it can give an improvement of 6.8% in the F1-score. Furthermore, we propose an adaptive tree-based ensemble network as the intrusion detection engine, primarily conducting accurate and efficient multiclass classification of network traffic data originating from both the IVNs and external network. To assess the efficacy of the proposed IDS, we use the network intrusion detection dataset CICIDS2017 and the IoV security dataset Car-Hacking. The experimental results involve a comprehensive comparison against the prevailing state-of-the-art techniques, revealing an impressive F1-score of 0.965 on the CICIDS2017 dataset and an even more remarkable F1-score of 0.9999 on the Car-Hacking dataset. These experimental outcomes tend to showcase superior prowess for multiclass classification on various cyber-attacks both from the external network and the IVNs in IoV.

However, there are certain limitations to our research that should be noted. We primarily focus on the detection of known threats, with a specific emphasis on achieving a highly accurate and efficient multiclass classification of network traffic in IoV. Nevertheless, our approach exhibits a comparatively lower capability in the detection of unknown or novel attacks. Furthermore, we have not delved deeply into the complexities associated with the deployment of the proposed IDS in real-world vehicular settings. These considerations will serve as critical areas of focus for our future research.

In future work, we would like to pay more attention to improving the model’s adaptability and resiliency to emerging threats. Additionally, we will conduct further investigations into the operational requirements for intrusion detection in IVNs and consider the practical usage of the IDS in this context.

## Figures and Tables

**Table 1:** A comparison of existing studies.

Categories	Methods	Relevant Model	Innovation/Challenge
IDSs for general networks	[13]	CSV-ISVM	Binary classification
	[14]	Genetic algorithms, KNN	Multiclass classification; slow training and high memory requirements
	[15]	K-Medoid clustering, SVM, Naïve Bayes classifier	Multiclass classification; need more research in determining optimal cluster numbers and initial cluster medoids
	[24,25,26,27,28,29,30,31]	DL-based model	DL models did not perform better than ML-based models in intrusion detection
IDSs for IoT and IoV	[16]	DT-based	Being able to fully detect four kinds of attacks and twenty-two other kinds of attacks; the model applicability to the IoV context
	[17,18,19,20,21]	ML-based model	distributed architectural approaches; the model applicability to the IoV context
	[22,23]	Federated learning	distributed architectural approach; preserving sensitive IoT information security
IDSs for IVNs	[12]	CNN	Datasets constructed from real vehicles; Limit to additional hardware
	[32]	DPFL-F2IDS	The challenge of striking a balance between utility and privacy metrics
	[33]	Statistical-based methods	Less accurate when multiple malicious events occur

**Table 2:** Class distribution of the CICIDS2017 training set after balancing.

Categories	Before Balancing	After Balancing
Benign	1,221,302	800,000
DoS/DDoS	192,161	450,000
PortScan	34,383	50,000
Brute Force	5131	50,000
Web Attack	1271	50,000
Botnet ARES	1166	55,000

**Table 3:** Impact on model detection performance using different approaches to reduce feature dimensionality.

Methods	F1-Score	Recall	Precision	Accuracy
Baseline	96.46	98.37	94.94	99.90
PCA	95.57	97.24	94.21	99.90
ML-based methods	96.36	98.65	94.61	99.90

**Table 4:** Detection performance of different models.

Models	F1-Score (%)	Recall (%)	Precision (%)	Accuracy (%)	Execution Time (ms)
RF	92.50	97.72	88.76	99.78	2.27 × 10^−4^
ET	92.68	98.11	89.18	99.79	2.23 × 10^−4^
XGBOOST	88.86	84.95	**99.36**	99.55	4.73 × 10^−4^
LIGHTGBM	91.54	**98.54**	86.52	99.03	0.0107
DT	95.74	97.57	94.22	99.86	1.56 × 10^−4^
XGBoost × 2 + RF + ET	94.77	92.30	98.66	99.88	2.66 × 10^−3^
ATBEN	**96.46**	98.37	94.94	**99.90**	3.91 × 10^−3^

**Table 5:** Performance evaluation on the CICIDS2017 dataset.

	F1-Score	Recall	Precision	Accuracy	Support
Benign	1.00	1.00	1.00	1.00	610,652
Botnet	0.73	0.91	0.81	0.96	583
Brute Force	1.00	1.00	1.00	1.00	2565
Dos/DDos	1.00	1.00	1.00	1.00	96,081
PortScan	0.98	1.00	0.99	1.00	17,192
Web Attack	0.98	0.99	0.99	0.99	635
Weighted Avg	1.00	1.00	1.00	-	727,708
Accuracy	1.00				727,708

**Table 6:** Performance evaluation on the Car-Hacking dataset.

	F1-Score	Recall	Precision	Accuracy	Support
DoS	1.00	1.00	1.00	1.00	726,995
Fuzzy	1.00	1.00	1.00	1.00	750,001
RPM	1.00	1.00	1.00	1.00	915,602
Gear	1.00	1.00	1.00	1.00	881,312
Norm	1.00	1.00	1.00	1.00	185,479
Weighted Avg	1.00	1.00	1.00	1.00	3,459,389
Accuracy	1.00				727,708

**Table 7:** Comparison between existing methods and ours on the CICIDS2017 dataset.

Methods	F1-Score	Recall	Precision	Accuracy	Category
MLP [46]	0.872	0.862	0.884	0.872	2
LSTM [46]	0.895	0.898	**0.984**	0.895	2
1D-CNN [46]	0.939	0.901	0.981	0.939	2
DeepGFL [48]	0.531	0.448	0.948	0.531	12
DBN [47]	0.940	**0.997**	0.887	0.940	6
Ours	**0.965**	0.984	0.949	**0.965**	6

**Table 8:** Comparison between existing methods and ours on the Car-Hacking dataset.

Methods	F1-Score (%)	Recall (%)	Precision (%)	Accuracy (%)
ET	99.96	99.96	99.96	99.97
RF	99.95	99.96	99.95	**99.99**
XGBOOST	72.46	69.72	80.73	75.53
LIGHTGBM	87.78	85.40	92.24	90.05
SVM [49]	93.3	98.3	95.7	96.5
KNN [49]	93.4	98.2	96.3	97.4
LSTM-AE [32]	99.0	99.9	99.0	99.0
DCNN [12]	99.91	99.84	99.84	99.93
HDL-IDS [50]	99.97	99.98	99.97	99.98
Ours	**99.99**	**99.99**	**99.99**	**99.99**

**Table 9:** A comparison of existing studies related to intrusion detection in IoV.

Methods	External Network Intrusion Detection	IVNs Intrusion Detection	Multiclass Classification	Feature Selection
[46]	✓			
[47]	✓		✓	
[49]		✓		
[50]	✓	✓		
[51]	✓			✓
[33]	✓			
[52]	✓			✓
Ours	**✓**	**✓**	**✓**	**✓**

## Data Availability

The datasets used in this paper are publicly available to everyone and can be accessed at: https://www.unb.ca/cic/datasets/ids-2017.html (accessed on 15 January 2022) and https://ocslab.hksecurity.net/Datasets/car-hacking-dataset (accessed on 20 December 2022).

## Appendix A

Denial-of-Service (DoS) attacks: In computing, a denial-of-service attack (DoS attack) is a cyber-attack in which the perpetrator seeks to make a machine or network resource unavailable to its intended users by temporarily or indefinitely disrupting the services of a host connected to a network. A denial-of-service attack is typically accomplished by flooding the targeted machine or resource with superfluous requests in an attempt to overload systems and prevent some or all legitimate requests from being fulfilled.

Web attacks: A web attack refers to any malicious activity or action taken by an individual, group, or automated script with the intention to compromise the security, functionality, or availability of a website, web application, or web server.

Sniffing attacks: Using sniffing tools, attackers can sniff sensitive information from a network, including email (SMTP, POP, IMAP), web (HTTP), FTP (Telnet authentication, FTP Passwords, SMB, NFS), and many more types of network traffic. The packet sniffer usually sniffs the network data without making any modifications in the network’s packets.

Portscan: A port scan or portscan is a process that sends client requests to a range of server port addresses on a host, with the goal of finding an active port, by which attackers can determine services available on a remote machine.

Brute-force attacks: A brute-force attack is a cryptanalytic attack that can, in theory, be used to attempt to decrypt any encrypted data (except for data encrypted in an information theoretically secure manner).

Botnet attacks: Botnet refers to the use of one or more propagation methods to infect a large number of hosts with bot programs, thereby forming a one-to-many controllable network between the controller and the infected hosts.

Fuzzy attacks: Injecting messages of totally random CAN ID and DATA values.

Spoofing attacks (RPM/gear): Injecting messages of certain CAN ID related to RPM/gear information.
